# Clinical Features and Prevalence of Osteosarcopenia in Chronic Pancreatitis

**DOI:** 10.5152/tjg.2025.24664

**Published:** 2025-05-05

**Authors:** Hüseyin Döngelli, Göksel Bengi, Raif Can Yarol, Canan Altay, Süleyman Dolu, Nilay Danış, Ömer Selahattin Topalak, Hale Akpınar, Servet Kızıldağ, Müjde Soytürk

**Affiliations:** 1 Department of Internal Medicine, Dokuz Eylül University Hospital, İzmir, Türkiye; 2 Department of Gastroenterology, Dokuz Eylül University Hospital, İzmir, Türkiye; 3 Department of Radiology, Dokuz Eylül University Hospital, İzmir, Türkiye; 4 Department of Medical Services and Techniques, Dokuz Eylül University Hospital, İzmir, Türkiye

**Keywords:** Chronic pancreatitis, osteoporosis, quality of life, sarcopenia, zinc deficiency

## Abstract

**Background/Aims:**

The prevalence of sarcopenia and osteopenia/osteoporosis is increased in chronic pancreatitis (CP). This study aims to evaluate the prevalence and related factors of osteosarcopenia in CP patients.

**Materials and Methods:**

Eighty-three CP patients were included in this cross-sectional observational study. Sarcopenia was assessed by measuring the surface area of the paravertebral muscles at the third lumbar region. Pancreatic fecal elastase (PFE) tests evaluated exocrine pancreatic insufficiency (EPI), while dual-energy x-ray absorptiometry scans assessed osteopenia/osteoporosis. EORTC PAN26 and a symptom questionnaire were administered, alongside nutritional marker assessments concurrent with PFE.

**Results:**

The prevalence of sarcopenia and osteopenia/osteoporosis was found to be 22.9% (n = 19) and 68.7% (n = 44), respectively. Factors associated with sarcopenia included male gender (OR: 4.9, *P* = .044), severe EPI (OR: 4.2, *P* = .043), smoking (OR: 4.6, *P* = .040), and zinc deficiency (OR: 2.2, *P* = .029). Severe EPI was significantly associated with osteopenia/osteoporosis (*P* = .010), diabetes mellitus (*P* = .001), sarcopenia (*P* = .016), and zinc deficiency (*P* = .012). Individuals with osteoporosis had higher PAN26 scores (*P* = .029). Factors independently associated with osteopenia/osteoporosis included female gender (OR: 7.8, *P* = .004), severe EPI (OR: 8.1, *P* = .003), and sarcopenia (OR: 5, *P* = .037). The prevalence of osteosarcopenia was 19.2%.

**Conclusion:**

Osteosarcopenia is common in CP patients. Smoking, zinc deficiency, EPI, and male gender are strongly associated with sarcopenia. Screening for osteosarcopenia is essential in CP patients to facilitate appropriate interventions.

**Main Points**

- The prevalence of osteosarcopenia is significantly elevated among individuals diagnosed with chronic pancreatitis (CP).
- Smoking, zinc deficiency, exocrine pancreatic insufficiency, and male gender are strongly associated with sarcopenia in patients with CP.
- Osteosarcopenia should be routinely screened in all patients with CP, and appropriate interventions should be implemented as necessary.

## Introduction

Chronic pancreatitis (CP) is a fibroinflammatory disease that progresses with the loss of both exocrine and endocrine parenchyma of the pancreas due to fibrosis resulting from chronic inflammation.^1^ Although chronic abdominal pain is the most common clinical feature in CP, long-term complications such as osteopenia/osteoporosis, exocrine pancreatic insufficiency (EPI), sarcopenia, and vitamin/mineral deficiencies are also observed in CP, which is a malnutrition syndrome.^2^ The diagnosis is often made between the ages of 34-54 with a male predominance. Alcohol is the most common cause of CP, and other etiological factors include genetic diseases (e.g., cystic fibrosis), autoimmune pancreatitis, obstructive causes, and recurrent severe acute pancreatitis.^3^^,^^4^

Sarcopenia is defined as a decrease in total body muscle mass with low muscle function.^5^ Although sarcopenia is associated with morbidity and mortality, especially in the geriatric population, it has also been associated with negative outcomes in diseases such as liver cirrhosis, malignancies, diabetes mellitus (DM), neurodegenerative disorders, and CP.^6^

The prevalence of sarcopenia is increased in CP patients compared to the normal population due to reasons such as chronic inflammation, limitation of physical activity due to pain, malnutrition, and chronic analgesic use.^7^

In a study by Olesen et al,^8^ the frequency of sarcopenia in CP patients was found to be 17%, and they found that sarcopenia was associated with a decrease in the general health of CP patients, an increase in the frequency of hospitalization, and a lower quality of life. In this study, EPI, smoking, and narcotic analgesic use were identified as risk factors for sarcopenia.

Exocrine pancreatic insufficiency is one of the most common complications of CP. In a study by Kempeneers et al,^9^ potential and definite EPI in CP patients were reported at 31% and 46%, respectively.

The prevalence of osteopenia/osteoporosis is high in patients with CP and may increase the risk of fractures. In a meta-analysis of 19 studies by Koh et al,^10^ it was shown that 19% of the patients diagnosed with CP were osteoporotic and 37% were osteopenic.

Osteosarcopenia is a newly defined syndrome that describes the co-occurrence of osteopenia/osteoporosis and sarcopenia.^11^ If it is not detected early and necessary precautions are not taken, it may lead to negative clinical consequences such as death, bone fractures, and deterioration in quality of life.^12^

The aim of the research is to evaluate the prevalence and related factors of osteosarcopenia in CP patients. It was hypothesized that sarcopenia is associated with osteopenia/osteoporosis in patients with CP. Another aim of the study was to identify factors associated with osteosarcopenia, such as comorbidity, analgesic habits, and serum nutritional markers.

## Materials and Methods

### Patients and Data Collection

A cross-sectional monocentric study in CP outpatients was conducted at the gastroenterology clinic of a university hospital, a tertiary care center. Research data were collected through patient files in hospital databases and outpatient clinic interviews. The study was approved by the Dokuz Eylül University’s non-interventional research ethics committee on July 28, 2021 with the approval number 2021/22-08.

Criteria for inclusion of patients in the study were defined as: being 18 years of age or older, having a diagnosis of CP, having cross-sectional imaging within the last 3 months, having no missing data, and volunteering to perform pancreatic fecal elastase (PFE). The hospital where the study was conducted was an endoscopic ultrasonography (EUS) center, and each patient was diagnosed with CP using the diagnostic criteria defined according to EUS findings.^13^

Exclusion criteria for patients from the study: not being within the recommended age range of 18 years of age or older, missing data, and having a diagnosis of malignancy, liver cirrhosis, or inflammatory bowel disease.

The study started with 136 patients, but 35 patients were excluded due to exclusion criteria. Due to incomplete data and patients who did not want to undergo the PFE test, 18 patients were not included in the study, and the study was conducted with 83 patients. Most of the missing data stemmed from the lack of computed tomography (CT) scans to evaluate sarcopenia, as well as the absence of laboratory findings such as zinc levels and 25-hydroxyvitamin D measurements.

Symptom questionnaires, anthropometric measurements, habits, medications, and comorbidities were also collected and recorded at the time the PFE was tested. Current smokers and regular drinkers were defined as tobacco or alcohol users. The etiology of CP was recorded according to the TIGAR-O (Toxic-metabolic, Idiopathic, Genetic, Autoimmune, Recurrent and severe acute pancreatitis, and Obstructive) classification.

### Laboratory Examinations and Nutritional Assessment

Body mass index (BMI) was calculated by dividing body weight by the square of height and was classified according to World Health Organization criteria: underweight (BMI <18.5 kg/m^2^), normal weight (BMI 18.5-24.9 kg/m^2^), overweight (BMI 25.0-29.9 kg/m^2^), and obese (BMI >30 kg/m^2^).

Complete blood count, serum amylase, lipase, B12, 25-OH vitamin D, ferritin, zinc, low-density lipoprotein, high-density lipoprotein, triglycerides, albumin, total protein, C-reactive protein, total bilirubin, and direct bilirubin were tested at the same time PFE was performed. Test results were recorded as numbers. Additionally, vitamin and mineral deficiencies were categorized as either absent or present. Deficiencies were defined using well-established thresholds from the literature.

### Sarcopenia Measurements

Abdominal CT images of the patients taken within the last 3 months were transferred to the workstation in the department of radiology at the institution (Philips Extended Brilliance Workspace V3.5.35.1011), which allows quantification of the tissue composition by using Hounsfield units (HU). Hounsfield unit (HU) thresholds for skeletal muscle, excluding viscera, ranged from −29 to +150. First, cross-sectional surface area measurements of the paraspinal muscles were taken at the level of the third lumbar vertebra (L3). Then, the paravertebral muscle index (PVMI) was calculated (cm^2^/m^2^) by dividing the muscle area by the square of the patient’s height.^14^ Radiological analysis for sarcopenia was performed by an experienced radiologist (R.C.Y. and C.A.) who was blinded to the patient results.

The cut-off values of PVMI for identifying sarcopenic patients were 26.3 cm^2^/m^2^ and 20.8 cm^2^/m^2^ for men and women, respectively. As there are no population-based PVMI threshold values established in Türkiye, thresholds from a previous study on a healthy cohort in Austria were used.^14^

### Exocrine Pancreatic Insufficiency

After obtaining informed consent, patients provided solid stool samples, which were analyzed for PFE enzyme levels using the ELISA method by experienced reseacher (S.K.). Since pancreatic enzyme replacement therapy (PERT) does not affect PFE results, patients continued PERT during stool sample collection. Only solid stool samples were accepted and stored at −20°C. After all samples were collected, PFE measurements were made only in the solid stool. Results were reported as µg/mL, and values above 200 µg/mL were considered to have no EPI. Pancreatic fecal elastase results below 200 µg/mL indicated EPI, with values between 100 and 200 µg/mL classified as mild and values below 100 µg/mL as severe.^15^

### Metabolic Bone Disease

Bone density for osteoporosis was assessed using dual-energy x-ray absorptiometry (DEXA). *Z* scores were evaluated for patients under 50 years of age, and T scores for patients over 50 years of age. Z scores below −2.5 indicated osteoporosis, scores between −2.5 and −1.5 indicated osteopenia, and scores above −1.5 were classified as normal.^16^

### Quality of Life Score

The European Organisation for Research and Treatment of Cancer (EORTC) PAN26 questionnaire was used to evaluate quality of life. Initially designed to assess the quality of life in pancreatic cancer patients, the PAN26 questionnaire has also been validated for CP patients.^17^ The EORTC PAN26 is the only accredited and reliable quality-of-life survey available in Turkish.^17^ In this survey, which consists of 26 questions and 4-level answers, the negative effects of the disease and treatments on the patient are evaluated. Responses are scored on a 4-point scale ranging from “not at all” to “very much.” All but 2 survey questions assess negative impacts. The exceptions (questions 53 and 54) assess satisfaction with health support and use a positive scale. To calculate the cumulative PAN26 score, responses to these 2 questions were adjusted by subtracting their values from 5 before inclusion.

### Statistical Analysis

All analyses were performed using the Statistical Package for Social Sciences (SPSS) 24.0 statistical package program (IBM SPSS Corp.; Armonk, NY, USA). Kolmogorov–Smirnov and Shapiro–Wilk tests were used for normality tests. Student’s *t*-test compared parametric continuous variables, while the Mann–Whitney *U* test analyzed non-parametric continuous variables. Results are presented as mean ± SD or median (min-max). In the comparison of categorical variables, Chi-Square test and Fisher’s exact test records were given, with the results presented as n (%). One-Way ANOVA compared parametric continuous variables, while the Kruskal–Wallis test was used for non-parametric variables. Results are presented as mean ± SD and median (min-max). Eta-squared analysis was used to evaluate effect size. Scheffe and Games tests were used for post hoc analyses. Binary logistic regression analysis was used to predict sarcopenia and osteoporosis, beginning with univariate analysis followed by multivariate modeling. Model fit was evaluated using Hosmer and Lemeshow tests, and statistical power was evaluated using Nagelkerke R Square. Statistical significance was defined as *P* < .05.

## Results

### Demographic and Clinical Characteristics of the Patients

The study initially included 136 patients; however, 35 were excluded due to exclusion criteria. An additional 18 patients were excluded due to missing data, leaving a total of 83 patients for analysis. The mean age of the 83 patients (37 women and 46 men) was 56.2 ± 13.6 years. The mean age at CP diagnosis was 49.7 ± 14.5 years. Of the 83 patients, 42 (50.6%) had a history of smoking, and 22 (26.5%) reported alcohol use. Among the comorbidities observed in the study population, 12 (14.5%) had no comorbid conditions. The most prevalent was DM, affecting 51.8% of participants, followed by hypertension at 45.8%. Coronary artery disease was present in 13.3%, chronic obstructive pulmonary disease (COPD) in 16.9%, hypothyroidism in 9.6%, and chronic kidney disease in 10.8% of individuals (Table 1).

In the symptom survey conducted at the time of diagnosis and/or prior to initiating PERT, abdominal pain was reported as the most common symptom by 71 patients (85.5%), whereas steatorrhea was the least common, reported by 13 patients.

The etiological classification based on the TIGAR-O framework revealed the following distribution among participants: toxic causes were the most common, identified in 36.1% of cases, including alcohol-related etiology (24.1%), hyperlipidemia (8.4%), and hypercalcemia (3.6%) as subcategories. Idiopathic cases accounted for 14.5%, while no cases were attributed to genetic causes. Autoimmune etiologies were noted in 19.3%, recurrent and severe cases in 24.1%, and obstructive causes in 6.0% of participants. When reviewing the past follow-ups of patients diagnosed with autoimmune pancreatitis, it was found that all had a history of corticosteroid use. However, in the survey conducted during the study, only 9 patients were actively using corticosteroids. These patients had been using corticosteroids for at least 1 year.

The most common complication was pancreatic ductal stones (10.8%), followed by common bile duct stenosis (8.4%), walled-off necrosis (WON) (7.2%), pseudocysts (6%), splenic vein thrombosis (6%), portal vein thrombosis (2.4%), and duodenal stenosis (1.2%) (Table 1).

Narcotic analgesics were classified as mild-, medium-, and heavy-acting, with usage rates of 26.5%, 4.8%, and 10.8%, respectively. The number of patients who underwent distal pancreatectomy was 16 (19.3%). The indications for pancreatectomy were pain palpation in 12 patients and suspicion of cancer/mass in 4 patients. When patients were evaluated based on predetermined threshold values for age and gender, hypoalbuminemia was detected in 16.9%, hypomagnesemia in 77.1%, zinc deficiency in 36.1%, vitamin D deficiency in 81.9%, vitamin B12 deficiency in 43.4%, and iron deficiency in 61.4%.

A total of 58 patients (69.9%) were undergoing PERT treatment. When comparing patients receiving PERT treatment with those not receiving it, no statistically significant difference was observed in vitamin D deficiency (82.8% vs. 80%, *P* = .763), zinc deficiency (36.2% vs. 36.0%, *P* = .986), osteoporosis (24.1% vs. 16.0%, *P* = .409), and sarcopenia (17.2% vs. 36%, *P* = .062).

### Prevalence of Sarcopenia and Association with Other Clinical Factors

Sarcopenia was more prevalent in men than in women (*P* = .019). Patients with sarcopenia were found to have lower BMI (*P* = .004). No significant relationship was observed between CP duration and sarcopenia. Smoking was significantly more common among patients with sarcopenia (*P* = .005). Although no statistically significant relationship was found between EPI and sarcopenia, the frequency of sarcopenia was observed to be higher in CP patients with severe EPI (*P* = .048).

Other biochemical tests showed no significant association between vitamin/mineral deficiencies and sarcopenia; however, zinc deficiency was significantly more common in patients with sarcopenia compared to those without (*P* = .024) (Table 2).

### Prevalence of Exocrine Pancreatic Insufficiency and Association with Other Clinical Factors

The average age of patients diagnosed with EPI was 57 years, with 27 women and 29 men. When EPI was classified as absent, mild, or severe, the frequency of osteopenia/osteoporosis increased significantly with the severity of EPI (*P* = .010). Although hypoalbuminemia, anemia, and vitamin and mineral deficiencies were more common in patients with EPI, only iron deficiency (*P* = .007) was statistically associated with EPI. Diabetes mellitus was associated with EPI (*P* = .001); additionally the duration of DM, HbA1c, and fasting blood glucose (FBG) levels were significantly higher in patients with severe EPI. There was no statistically significant difference in zinc deficiency when EPI was evaluated as mild/severe versus no EPI (35.7% vs. 37%, *P* = .906). However, a statistically significant difference in zinc deficiency was observed when EPI was evaluated as severe versus mild/no EPI (50% vs. 25.5%, *P* = .021). Moreover, zinc deficiency was more frequently observed in patients with severe EPI (*P* = .012) when EPI was assessed ordinally as severe, mild, and no EPI (Table 3).

### Prevalence of Osteoporosis and Association with Other Clinical Factors

In the cohort of 83 individuals, 18 had osteoporosis and 42 had osteopenia. Osteoporosis was detected in 13 female and 5 male patients, with this difference being statistically significant (*P* = .008).

The cumulative results of the PAN26 quality of life survey were significantly worse in individuals with osteoporosis (*P* = .029) (Table 4). This finding indicates that patients with osteoporosis have a lower quality of life compared to those without.

### Regression Analysis for Sarcopenia and Osteopenia/Osteoporosis

To predict sarcopenia using regression analysis, the variables listed in the table below were first evaluated individually and then multivariately. In univariate analysis, male gender, smoking, severe EPI, and zinc deficiency were determined to be associated with sarcopenia. These variables were then evaluated by multiple regression analysis; severe EPI and smoking were found to be independently associated with sarcopenia (Table 5).

A logistic regression analysis was conducted to identify factors associated with osteopenia/osteoporosis. In the univariate analysis, female sex, severe EPI, and age were identified as factors associated with osteopenia/osteoporosis. These variables were subsequently analyzed using multiple regression analysis. Female sex, severe EPI, age, and sarcopenia were found to be independently associated with osteopenia/osteoporosis (Table 6).

## Discussion

The incidence of CP is increasing, placing a significant burden on society and the healthcare system due to chronic pain and its associated complications. In this study, the prevalence of sarcopenia and osteopenia/osteoporosis in CP patients and their negative impact on patient outcomes were investigated. Among the 83 patients included in the study, sarcopenia was observed in 19 (22.9%) and osteoporosis in 18 (21.7%). Male sex, severe EPI, and smoking were found to be independently associated with sarcopenia. Female sex, severe EPI, age, and sarcopenia were found to be independently associated with osteopenia/osteoporosis. The pancreras (PAN) 26 survey demonstrated that osteopenia/osteoporosis negatively impacts patients’ quality of life. A strong association was observed between sarcopenia and osteopenia/osteoporosis.

Factors involved in etiology can be determined by the TIGAR-O classification system.^18^ In this study, the most common etiological group was toxic causes (36%), followed by recurrent pancreatitis (20%), autoimmune pancreatitis (16%), idiopathic causes (14%), and obstructive causes (5%). A study involving 1071 patients in Northern Europe found that the most common etiological factor was toxic (alcohol, cigarettes, etc.) at 55%, followed by 12% idiopathic, 10% hereditary, 8% autoimmune, and 6% obstructive causes.^19^ Since the study was single-center, no rare genetic causes were found in the study group; however, 2 patients who were excluded due to exclusion criteria had genetic origins. Among the causes, autoimmune pancreatitis was more common, likely because the study center was a tertiary center where EUS was performed.

The most common complications were pancreatic ductal stones (10.8%), followed by common bile duct stenosis (8.4%), WON (7.2%), pseudocyst (6%), splenic vein thrombosis (6%), portal vein thrombosis (2.4%), and duodenal stenosis (1.2%). In this study, pancreatic carcinoma was excluded based on the study design. Pancreatic DM was not presented as a complication due to the difficulty of evaluating it separately. A single-center study conducted in Türkiye in 2016 on 168 CP patients found that 34% had pseudocysts, 21% had common bile duct stenosis, 13% had splenic vein thrombosis, 2% had pancreatic cancer, and 2% had duodenal stenosis.^20^ The lower complication rates in the study can be attributed to the fact that only current clinical and imaging findings from the year of the study were evaluated. In contrast, the other study was retrospective and screened complications in patients over a 5-year period. Although complication rates were lower, the order of prevalence remained similar.

Although the parameters frequently used in sarcopenia research are skeletal muscle mass index (SMI) or psoas muscle index measurements at the L3 vertebra level, PVMI was used in the study. There are publications stating that PVMI measurements are more useful in showing the frequency of sarcopenia in liver cirrhosis and cancer patients, as well as the effect of sarcopenia on prognosis. However, to the best of knowledge, there is no study that measures sarcopenia using PVMI in CP patients.^14^^,^^21^

In this study, univariate regression analysis revealed that male gender, severe EPI, smoking, and zinc deficiency increase the risk of sarcopenia. Furthermore, multivariate regression analysis showed that smoking, male gender, and severe EPI independently increased the risk of sarcopenia by over 4 times. It is believed that severe EPI is one of the most important causes of malnutrition and therefore poses a risk for sarcopenia. Smoking and alcohol use contribute to the development of sarcopenia in multiple ways, such as increased oxidative stress, negative effects on the vessels, and increased malnutrition.^22^ In the study by Olesen et al,^8^ which investigated the risk factors for sarcopenia in CP patients, EPI, smoking, and narcotic analgesic use were identified as risk factors, and these findings are consistent with our study. In the study by Shintakuya et al,^23^ involving 132 CP patients, a strong relationship was found between EPI and sarcopenia using SMI and the 13C-triglyceride breath test at the L3 vertebra level. In contrast, the findings showed a relationship only between severe EPI, not mild or moderate EPI, and sarcopenia, likely due to differences in EPI evaluation methods. Another study investigating the relationship between mineral deficiencies and sarcopenia showed a link between functional sarcopenia (low walking speed) and zinc deficiency. To the best of knowledge, this study is the first to demonstrate the relationship between sarcopenia, as measured by cross-sectional imaging methods, and zinc deficiency in CP patients.^24^ Zinc is an important mineral that plays a role in muscle formation and cell energy metabolism. Therefore, zinc deficiency increases the tendency to develop sarcopenia.^25^

Mild and severe EPI were detected in 20 (24.1%) and 36 (43.4%) of the patients included in the study, respectively. In a study involving 430 CP patients, the presence of EPI was shown to independently increase mortality, highlighting the importance of EPI screening in CP patients.^26^ Among patients with EPI in our study, 71.4% had iron deficiency, and 60% of those with zinc deficiency had severe EPI. Additionally, a strong association was found between DM and EPI, with 36 DM patients also diagnosed with EPI. Parameters related to DM, including DM duration, HbA1c, FBG, and insulin use, were closely linked to EPI. In a study by Pan et al,^27^ EPI was identified as a risk factor for DM in CP patients. While the diagnostic criteria for pancreatic diabetes (type 3c) are not fully established, the presence of EPI is generally considered necessary for diagnosis.^28^ Furthermore, it was observed that a significant correlation between increasing severity of EPI and higher rates of osteopenia/osteoporosis (*P* = .019).

Osteopenia and osteoporosis are other critical complications of CP. In this cohort, osteopenia was detected in 42 patients (50.6%), and osteoporosis in 18 patients (21.7%). Of those with osteoporosis, 72% (13/18) were female. The risk of bone fractures in CP patients is 7 times higher than in the general population.^29^ Contributing factors include smoking, alcohol use, female gender, age, chronic inflammation, calcium/phosphorus malabsorption, sedentary lifestyle due to pain, vitamin D deficiency, low sun exposure, and EPI.^29^ Osteoporosis independently increases mortality in CP patients.^30^ In this study, individuals with osteoporosis had higher PAN26 scores, indicating a lower quality of life. Univariate regression analysis showed significant associations between female gender, increasing age, and severe EPI with osteopenia/osteoporosis. However, no significant relationship was observed with smoking, alcohol use, or vitamin D deficiency. Multivariate logistic regression analysis identified female gender, sarcopenia, and severe EPI as the most important factors associated with osteopenia/osteoporosis. The P-BONE study, a prospective analysis of 211 CP patients, reported EPI in 58%, osteoporosis in 22%, osteopenia in 42%, and vitamin D deficiency in 58%. Female gender, older age, and low BMI were significant risk factors for osteoporosis.^31^ These findings align with the study results, although sarcopenia and severe EPI were additionally identified as potential risk factors for osteopenia/osteoporosis. Interestingly, the lack of a significant relationship between osteopenia/osteoporosis and vitamin D deficiency in this study, despite its known role as a key risk factor, may be due to the high prevalence of vitamin D deficiency among CP patients overall. Finally, osteosarcopenia, a condition where sarcopenia and osteoporosis coexist, was detected in 16 patients (19.2%).

The study has some limitations. First, the study population comprised a relatively small cohort of patients from a single center, limiting the generalizability of the findings. Longitudinal, multicenter, and prospective studies are necessary to confirm whether the factors identified as associated with osteosarcopenia genuinely increase the risk. Secondly, the PVMI method used for sarcopenia diagnosis lacks a validated threshold value specific to the Turkish population. Population-based studies are needed for appropriate PMVI thresholds in Türkiye. Third, only muscle mass was evaluated in this study. Functional muscle strength tests, such as hand grip or up-and-go tests, were not included in defining sarcopenia, which may have influenced the results. Fourth, in the study, EUS was used for the diagnosis of CP, and CT was utilized for identifying sarcopenia. However, a comprehensive evaluation incorporating pancreatic findings from EUS, endoscopic retrograde cholangiopancreatography (ERCP), and cross-sectional imaging together with osteosarcopenia would have provided more interesting and valuable insights. Lastly, this study is a cross-sectional, single-center study. Larger, multicenter, and prospectively designed studies are needed to elucidate the true relevance of these findings.

In conclusion, osteosarcopenia has a notably high prevalence among patients with CP. A significant relationship exists between osteopenia/osteoporosis and sarcopenia in patients with CP. Osteopenia/osteoporosis substantially reduces the quality of life in patients with CP. These findings underscore the necessity of investigating sarcopenia and osteopenia/osteoporosis in CP patients and highlight the importance of early intervention and treatment for osteosarcopenia.

## Figures and Tables

**Table 1.** Demographic and Clinical Characteristics of Patients

	Total (n : 83)
Age mean ± SD (min-max)	56.2 ± 13.6 (20-83)
Gender n (%) Female Male	37 (44.6) 46 (55.4)
CP duration mean ± SD (min-max)	6.5 ± 3.4 (2-17)
Age at diagnosis mean ± SD (min-max)	49.7 ± 14.5 (17-81)
PERT n (%)	58 (69.9)
BMI mean ± SD (min-max)	25.16 ± 4.02 (16-37)
Smoking n (%)	42 (50.6)
Alcohol n (%)	22 (26.5)
Comorbidities n (%) None Diabetes mellitus Hypertension Coronary artery disease COPD Hypothyroidism Chronic kidney disease	12 (14.5) 43 (51.8) 38 (45.8) 11 (13.3) 14 (16.9) 8 (9.6) 9 (10.8)
DM duration mean ± SD (min-max)	5.4 ± 7.2 (0-30)
HbA1c mean ± SD (min-max)	6.9 ± 1.8 (5-14)
FBG mean ± SD (min-max)	126.5 ± 58.8 (60-400)
Insulin use n (%)	26 (31.3)
Paracetamol n (%)	65 (78.3)
NSAID n (%)	47 (56.6)
Narcotic analgesic n (%) None Mild (Codeine) Medium (Tramadol) Heavy (Meperidine, Oxycodone, Morphine, Fentanyl)	48 (57.8) 22 (26.5) 4 (4.8) 9 (10.8)
Bone density measurement n (%) Normal Osteopenia Osteoporosis	23 (27.7) 42 (50.6) 18 (21.7)
Pancreatic fecal elastase mean ± SD (min-max)	193.59 ± 204.29 (15-761)
Exocrine pancreatic insufficiency n (%) None (PFE > 200 μg/g) Mild (100 < PFE<200 μg/g) Severe (PFE < 100 μg/g)	27 (32.5) 20 (24.1) 36 (43.4)
Sarcopenia n (%)	19 (22.9)
Osteosarcopenia n (%)	16 (19.2)
Lipase mean ± SD (min-max)	65.78 ± 62.55 (3-265)
Amylase mean ± SD (min-max)	85.17 ± 55.11 (13-318)
PAN 26 cumulative score mean ± SD (min-max)	52.49 ± 13.27 (26-88)
Symptoms n (%) Abdominal pain Bloating Nausea Constipation Diarrhea Dyspepsia Steatorrhea	71 (85.5) 49 (59) 45 (54.2) 24 (28.9) 24 (28.9) 22 (26.5) 13 (15.7)
Etiology (TIGAR-O) n (%) Toxic Alcohol Hyperlipidemia Hypercalcemia Idiopathic Genetic Autoimmune Recurrent and severe Obstructive	30 (36.1) 20 (24.1) 7 (8.4) 3 (3.6) 12 (14.5) 0 (0) 16 (19.3) 20 (24.1) 5 (6.0)
Complications n (%) None Pancreatic ductal stone Common bile duct stenosis Pancreatic duct stenosis WON Splenic vein thrombosis Pseudocyst Portal vein thrombosis Stomach/Duodenum stenosis	42 (50.6) 9 (10.8) 7 (8.4) 6 (7.2) 6 (7.2) 5 (6.0) 5 (6.0) 2 (2.4) 1 (1.2)

BMI, body mass index; COPD, chronic obstructive pulmonary disease; CP, chronic pancreatitis; DM, diabetes mellitus; FBG, fasting blood glucose; NSAID, non-steroid anti-inflammatory drug; PERT, pancreatic enzymes replacement therapy; PFE, pancreatic fecal elastase; WON, walled-off necrosis.

**Table 2.** Patients with Normal Skeletal Muscle Mass Compared to Sarcopenic Patients

	Sarcopenia (n = 19)	No Sarcopenia (n = 64)	*P*
Age mean ± SD	57.8 ± 9.6	55.7 ± 14.6	.548
Gender n (%) Female Male	4 (21.1) 15 (78.9)	33 (51.6) 31 (48.4)	**.019**
BMI mean ± SD	22.84 ± 3.35	25.85 ± 3.97	**.004**
CP duration mean ± SD	6.8 ± 3.9	6.4 ± 3.2	.706
Smoking n (%) Yes No	15 (78.9) 4 (21.1)	27 (42.2) 37 (57.8)	**.005**
Alcohol use n (%)	8 (42.1)	14 (21.9)	.079
BMI groups n (%) Underweight Normal weight Overweight Obese	2 (10.5) 9 (47.4) 8 (42.1) 0 (0)	1 (1.6) 24 (37.5) 31 (48.4) 8 (12.5)	.106
Narcotic analgesic n (%) None Mild (Codeine) Medium (Tramadol) Heavy (Morphine etc.)	8 (42.1) 5 (26.3) 1 (5.3) 5 (26.3)	40 (62.5) 17 (26.6) 3 (4.7) 4 (6.3)	.089
Osteopenia/osteoporosis n (%) Osteoporosis Osteopenia Normal	6 (31.6) 10 (52.6) 3 (15.8)	12 (18.8) 32 (50) 20 (31.3)	.302
EPI n (%) Yes No	12 (63.2) 7 (36.8)	44 (68.8) 20 (31.2)	.648
Severe EPI n (%) Yes No	12 (63.2) 7 (36.8)	24 (37.5) 40 (62.5)	**.048**
Zinc deficiency n (%)	11 (57.9)	19 (29.7)	**.025**
Lipase mean ± SD	64.8 ± 53.1	66.0 ± 65.4	.944
Amylase mean ± SD	98.1 ± 61.8	81.3 ± 52.8	.244
PAN26 cumulative score mean ± SD	54.2 ± 16.5	51.9 ± 12.2	.524

BMI, body mass index; CP, chronic pancreatitis; EPI, exocrine pancreatic insufficiency.

**Table 3.** EPI (No-Mild-Severe) and its Association with Other Clinical Parameters

	No EPI (n = 27)	Mild EPI (n = 20)	Severe EPI (n = 36)	*P*
Age mean ± SD	55.4 ± 13.9	57.8 ± 13.7	55.9 ± 13.7	.802
Gender n (%) Female Male	10 (37) 17 (63)	11 (55) 9 (45)	16 (44.4) 20 (55.6)	.472
BMI mean ± SD	25.96 ± 4.15	26 ± 4.12	24.1 ± 3.72	.229
CP duration mean ± SD	5.6 ± 2.7	6.1 ± 3.1	7.4 ± 3.8	.109
Osteopenia/osteoporosis n (%) Yes No	15 (55.6) 12 (44.4)	13 (65) 7 (35)	32 (88.9) 4 (11.1)	**.010**
Steatore n (%)	4 (4.8)	1 (1.2)	8 (9.6)	.234
Narcotic analgesic use n (%) None Mild (Codeine) Medium (Tramadol) Heavy (Morphine etc.)	19 (70.4) 5 (18.5) 2 (7.4) 1 (3.7)	13 (65) 5 (25) 0 (0) 2 (10)	16 (44.4) 12 (33.3) 2 (5.6) 6 (16.7)	**.030**
Sarcopenia n (%) Yes No	7 (25.9) 20 (74.1)	0 (0) 20 (100)	12 (33.3) 24 (66.6)	**.016**
Insulin use n (%)	3 (11.1)	7 (35)	16 (44.4)	**.017**
DM n (%)	7 (25.9)	13 (65)	23 (63.8)	**.005**
Alcohol use n (%)	7 (25.9)	4 (20)	11 (30.6)	.690
Smoking n (%)	14 (51.9)	8 (40)	20 (55.6)	.530
Lipase mean ± SD	76.5 ± 56.9	55.9 ± 55.2	63.2 ± 70.3	.122
Amylase mean ± SD	91.7 ± 52.5	81.9 ± 54.2	82.0 ± 58.4	.540
Zinc deficiency n (%)	10 (37)	2 (10)	18 (50)	**.012**
Iron deficiency n (%)	11 (13.3)	15 (18.1)	25 (30.1)	**.025**
PAN26 score mean ± SD	51.3 ± 10.9	51.0 ± 13.2	54.1 ± 14.9	.785

BMI, body mass index; CP, chronic pancreatitis; DM, diabetes mellitus; EPI, exocrine pancreatic insufficiency.

**Table 4.** Osteoporosis and its Association with Other Clinical Parameters

	Osteoporosis (n = 18)	No Osteoporosis (n = 65)	*P*
Age mean ± SD	61.0 ± 12.4	54.9 ± 13.7	.090
Gender n (%) Female Male	13 (72.2) 5 (27.8)	24 (36.9) 41 (63.1)	**.008**
BMI mean ± SD	25 ± 4	25 ± 4	.494
CP duration mean ± SD	7.4 ± 3.7	6.2 ± 3.3	.167
Smoking n (%)	8 (44.4)	34 (52.3)	.555
Alcohol use n (%)	3 (16.7)	19 (29.2)	.285
BMI Groups n (%) Underweight Normal weight Overweight Obese	0 (0) 6 (33.3) 9 (50) 3 (16.7)	3 (4.6) 27 (41.5) 30 (46.2) 5 (7.7)	.514
Sarcopenia n (%)	6 (33.3)	13 (20)	.340
EPI n (%)	13 (72.2)	43 (66.2)	.627
Vitamin D deficiency n(%)	15 (83.3)	53 (81.5)	.861
Zinc deficiency n (%)	7 (38.9)	23 (35.4)	.784
Lipase mean ± SD	47.6 ± 54.0	70.8 ± 64.1	.165
Amylase mean ± SD	79.0 ± 61.1	86.8 ± 53.6	.595
PAN26 cumulative score mean ± SD	57.7 ± 10.1	51 ± 13.7	**.029**

BMI, body mass index; CP, chronic pancreatitis; EPI, exocrine pancreatic insufficiency.

**Table 5.** Logistic Regression Analysis of Sarcopenia Related Factors

	Univariate Analysis			Multivariate Analysis		
	OR	95% CI	*P*	OR	95% CI	*P*
Age	1.01	0.97-1.05	.544	1.02	0.96-1.08	.531
Male sex	3.99	1.19-13.34	**.025**	4.08	1.01-16.66	**.050**
Severe EPI	2.85	0.98-8.25	.052	2.61	1.04-8.71	**.048**
Smoking	5.13	1.53-17.22	**.008**	4.03	1.07-15.13	**.040**
Zinc deficiency	3.25	1.13-9.37	**.029**	3.08	0.90-10.48	.072
DM	1.65	0.58-4.67	.338			
Lipase	1	0.99-1.00	.994			
Amylase	0.99	0.98-1.00	.252			

DM, diabetes mellitus; EPI, exocrine pancreatic insufficiency; OR, odds ratio.

**Table 6.** Logistic Regression Analysis of Osteopenia/Osteoporosis Related Factors

	Univariate Analysis			Multivariate Analysis		
	OR	95% CI	*P*	OR	95% CI	*P*
Age	0.95	0.92-0.99	**.027**	0.93	0.88-0.98	**.013**
Female sex	4.11	1.35-12.52	**.013**	7.89	1.96-31.78	**.004**
Severe EPI	5.42	1.64-17.87	**.005**	8.13	1.99-33.13	**.003**
Sarcopenia	2.42	0.63-9.27	.196	5.05	1.10-23.13	**.037**
DM	2.62	0.96-7.13	.059	1.21	0.35-4.18	.762
Smoking	1.09	0.41-2.85	.859			
Alcohol use	1.31	0.45-3.80	.616			
Vitamin D deficiency	2	0.62-6.44	.245			
Lipase	1.00	0.99-1.01	.127			
Amylase	1.00	0.99-1.01	.560			

DM, diabetes mellitus; EPI, exocrine pancreatic insufficiency; OR, odds ratio.

## Data Availability

The data that support the findings of this study are available upon request from the corresponding author.
